# Finding invisible quantitative trait loci with missing data

**DOI:** 10.1111/pbi.12942

**Published:** 2018-05-28

**Authors:** Iulian Gabur, Harmeet S. Chawla, Xiwei Liu, Vinod Kumar, Sébastien Faure, Andreas von Tiedemann, Christophe Jestin, Emmanuelle Dryzska, Susann Volkmann, Frank Breuer, Régine Delourme, Rod Snowdon, Christian Obermeier

**Affiliations:** ^1^ Department of Plant Breeding Justus Liebig University Giessen Germany; ^2^ IGEPP, INRA, AGROCAMPUS OUEST Univ Rennes Le Rheu France; ^3^ Biogemma Mondonville France; ^4^ Section of General Plant Pathology and Crop Protection Georg August University Göttingen Germany; ^5^ Terres Inovia Thiverval‐Grignon France; ^6^ Syngenta Seeds France SAS Saint‐Sauveur France; ^7^ KWS SAAT SE Einbeck Germany

**Keywords:** *Brassica napus*, quantitative resistance, presence–absence variation, single nucleotide absence polymorphism, SNaP

## Abstract

Evolutionary processes during plant polyploidization and speciation have led to extensive presence–absence variation (PAV) in crop genomes, and there is increasing evidence that PAV associates with important traits. Today, high‐resolution genetic analysis in major crops frequently implements simple, cost‐effective, high‐throughput genotyping from single nucleotide polymorphism (SNP) hybridization arrays; however, these are normally not designed to distinguish PAV from failed SNP calls caused by hybridization artefacts. Here, we describe a strategy to recover valuable information from single nucleotide absence polymorphisms (SNaPs) by population‐based quality filtering of SNP hybridization data to distinguish patterns associated with genuine deletions from those caused by technical failures. We reveal that including SNaPs in genetic analyses elucidate segregation of small to large‐scale structural variants in nested association mapping populations of oilseed rape (*Brassica napus*), a recent polyploid crop with widespread structural variation. Including SNaP markers in genomewide association studies identified numerous quantitative trait loci, invisible using SNP markers alone, for resistance to two major fungal diseases of oilseed rape, *Sclerotinia* stem rot and blackleg disease. Our results indicate that PAV has a strong influence on quantitative disease resistance in *B. napus* and that SNaP analysis using cost‐effective SNP array data can provide extensive added value from ‘missing data’. This strategy might also be applicable for improving the precision of genetic mapping in many important crop species.

## Introduction

Structural variation in genomes of humans, animals and plants is an essential class of genetic polymorphism that is today commonly used for genomic analysis (Dolatabadian *et al*., 2017). Common forms of short and longer range structural variation include small insertions and deletions (InDels), copy number variation (CNV) and presence–absence variation (PAV). Traditionally, InDels have been defined as short presence–absence polymorphisms spanning from 1 to 50 bp, whereas CNV results from gain or losses of larger DNA segments ranging from a few nucleotides to several kb of DNA in the size range of genes (reviewed in Saxena *et al*., 2014; Żmieńko *et al*., 2014). An extreme form of CNV is characterized by deletions of DNA sequences in one or more individuals of a population, which is also termed PAV (Saxena *et al*., 2014).

In recent years genetic diversity for structural genome variation in the form of InDels, CNV and PAV have been investigated widely in humans (Iafrate *et al*., 2004), bacteria (Arrach *et al*., 2008), animals (Graubert *et al*., 2007; Guryev *et al*., 2008; Snijders *et al*., 2005; Wilson *et al*., 2006) and plants (Batley *et al*., 2003; Hurgobin *et al*., 2017; Qian *et al*., 2016; Schiessl *et al*., 2017; Springer *et al*., 2009; Stein *et al*., 2017). In crops, PAV has been attributed to evolutionary processes associated with natural selection and breeding (Hurgobin *et al*., 2017; Żmieńko *et al*., 2014). Completion of reference genomes for most major crops and rapidly decreasing prices for genotyping‐by‐sequencing (GBS) have facilitated identification of PAV on a whole‐genome level. On the other hand, detection of PAV in GBS and skim‐sequencing datasets can be complicated by difficulties in distinguishing genuine deletions from regions with insufficient sequence coverage, along with bioinformatic challenges associated with haplotype imputation.

Gene CNV has been implicated in the control of many agronomic traits in different crop species, for example flowering time and plant height in *Brassica napus*, oilseed rape (Schiessl *et al*., 2017), ancestral evolution events and domestication in maize (Springer *et al*., 2009), or vernalization and winter hardiness in wheat (Würschum *et al*., 2015, 2017). CNV is also involved in resistance against pathogens, with nucleotide‐binding leucine‐rich repeat (NB‐LRR), thaumatin‐like protein (TLP) and receptor‐like kinase (RLK) genes being commonly involved in local gene duplications leading to variable copy number (Saxena *et al*., 2014).

*Brassica napus* (oilseed rape, canola, kale, rutabaga/swede) is a recent allopolyploid crop species that arose from interspecific hybridization between two diploid progenitors, *B. oleracea* and *B. rapa* (Snowdon, 2007), and rapidly acquired vast ecogeographic and agronomic diversity that ultimately led to its establishment as a globally important crop. Post polyploidization homoeologous exchanges during meiosis between chromosomes of the A and C subgenomes have been identified as a major driver of genome diversity in *B. napus* (Chalhoub *et al*., 2014; Hurgobin *et al*., 2017; Samans *et al*., 2017). Homoeologous rearrangements, including gene conversions (Chalhoub *et al*., 2014), CNV (Schiessl *et al*., 2017), PAV and segmental deletions (Hurgobin *et al*., 2017; Samans *et al*., 2017), underlie widespread structural and functional genome variation in both natural *B. napus* forms and in *de novo*, synthetic *B. napus* accessions. All these recent studies demonstrated that in *B. napus* gene terms associated with plant resistance and stress responses are strongly enriched among genes affected by deletions due to homoeologous exchanges, suggesting that PAV might be an important mechanism in crop disease resistance.

Major diseases of oilseed rape are caused by fungal pathogens transmitted by airborne or soil‐borne spores. In comparison with cereal crops, where gene‐for‐gene resistance interactions with important fungal virulence genes (e.g. for rust and mildew diseases) play an important role in breeding for crop protection, the most effective adult‐plant resistances to fungal diseases in oilseed rape are quantitative in nature. Vastly increased production, shorter crop rotations and global warming have led to particularly strong disease pressure in major growing areas for the fungal pathogens *Verticillium longisporum*,* Sclerotinia sclerotiorum* and *Leptosphaeria maculans* (Barbetti *et al*., 2012; Siebold and von Tiedemann, 2012). Among these, blackleg disease (Phoma stem canker) caused by *L. maculans* (anamorph *Phoma lingam*) is the most economically important disease of oilseed rape in Europe, North America and Australia, while Sclerotinia stem rot caused by the necrotrophic pathogen *S. sclerotiorum* causes substantial yield losses in all major growing areas throughout Europe, Australia, North America and China (Delourme *et al*., 2011). Major genes for resistance to blackleg disease (reviewed by Delourme *et al*., 2011) are effective at seedling stage but more durable when used in association with quantitative, adult‐plant resistance. No major‐gene resistance to *S*. *sclerotiorum* is available in *B. napus* so that identification and combination of quantitative genetic resistance factors are essential for breeding.

Resources for high‐throughput genomics are today broadly implemented for Brassica crops. Reference genome sequences for *B. rapa* (The *Brassica rapa* Genome Sequencing Project Consortium, 2011); *B. oleracea* (Liu *et al*., 2014) and *B. napus* (Chalhoub *et al*., 2014) have been supplemented by large‐scale resequencing (Schmutzer *et al*., 2015) or transcriptome datasets (He *et al*., 2015). As in many other major crops, one of the most broadly used tools for genetic analysis in *Brassica* crops is a community‐designed, high‐density single nucleotide (SNP) genotyping array (Clarke *et al*., 2016; Mason *et al*., 2017). It has been extensively applied for high‐density genetic mapping and QTL analysis (e.g. Liu *et al*., 2013; Luo *et al*., 2017; Wang *et al*., 2015), genomewide association studies for a wide range of traits (e.g. Hatzig *et al*., 2015; Li *et al*., 2014, 2016; Schiessl *et al*., 2015; Sun *et al*., 2016; Wan *et al*., 2017; Xu *et al*., 2016) and genomic selection (Jan *et al*., 2016; Zou *et al*., 2016).

One objective of this study was to improve the resolution of QTL mapping for fungal disease resistance in *B. napus* using high‐density SNP array data. We also elucidate the relevance and role of small‐scale and large‐scale PAV in the *B. napus* genome in relation to disease resistance. Data filtering approaches were designed to identify presence–absence variants in high‐density SNP array data and include these ‘missing’ data as an additional dimension in genomewide association studies.

## Results

### From failed SNP calls to single nucleotide absence polymorphism markers

**Figure 1:**
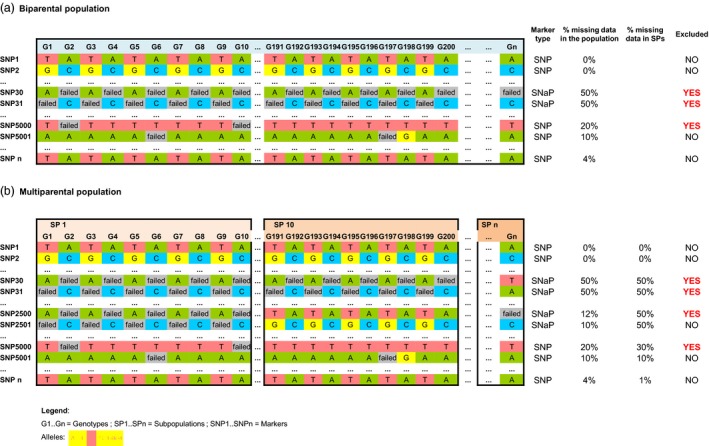
Schematic representation of allele segregation patterns and frequencies within and across subpopulations for different single nucleotide polymorphism (SNP) types observed in homozygous biparental (a) or multiparental mapping populations (b) and SNP probes excluded from analysis applying standard filtering procedures.

Failed SNP calls are commonly observed in genotyping experiments applying chip hybridization technologies. Thus, markers which show excessive frequencies of failed calls are often removed from genotyping matrices for downstream analyses (Mason *et al*., 2017). Although failed SNP calls are expected to be due to technical artefacts, in case of genuine deletions they may also represent biologically useful information due to potential association with gene PAV. We distinguished failed SNP calls representing single nucleotide absence polymorphisms (SNaP) from random technical failures in raw SNP chip data by filtering for segregation patterns in a multiparental homozygous mapping population. In an inbred or homozygous biparental mapping population, a segregation allele frequency of 50% is expected for a SNaP (e.g. A:failed, or C:failed in Figure 1a for SNP30 and SNP31), because the presence‐allele will be amplified only from one parental line and is absent in the other parental line. In our multiparental population, five diverse parents were crossed with one common parent so that a SNaP has an expected frequency of failed calls within each subpopulation of 50%, whereas the expected frequency of failed calls across the total population will be 10% if the absence derives from (only) one of the diverse parents. A threshold of 10% is usually used in the standard filtering approach as failed SNP call frequency to exclude markers from further analyses, thus eliminating potential SNaP markers.

According to the standard filtering approach as described in experimental procedures, 18 068 polymorphic SNP markers (63.5% of a total of 28 073 anchored markers) were selected for SNP‐based genomewide association studies (GWAS). In a refined filtering approach, a three‐step method was used to select SNaP markers that could indicate structural presence–absence polymorphisms.

Many of the 36.5% SNP markers removed during this standard filtering procedure showed a low frequency of failed calls in the 5 nested association mapping (NAM) subpopulations. However, separate investigation of each subpopulation revealed SNaP frequencies that were frequently close to the expected segregation pattern of a biparental homozygous population (50%). Figure 1b shows an example of different observed SNP marker patterns within and across subpopulations (SP1 = subpopulation 1, SP10 = subpopulation 10), which would be excluded from analysis using common filtering approaches. For example, markers SNP30 and SNP31 show a dominant A/failed or C/failed allele pattern with ~50% frequency across all subfamilies, because the absence derives from the recurrent parent, whereas SNP2500 and SNP2501 show ~50% A/failed or C/failed in only one subfamily because the absence derives from only one of the 5 diverse parents. All 10 005 excluded SNPs were reanalysed for specific segregation patterns within the five subpopulations, using an allele frequency threshold of 15%–85% between failed/present calls and considering segregation distortion. SNaP markers identified by segregation patterns were validated by physical positioning in the reference genome and identification of neighbouring SNP loci which showed corresponding SNaP patterns in the same genotypes. Using this approach, a number of 3627 SNaPs were identified, recovered and included in subsequent GWAS.

The mean genomic distance between polymorphic markers improved from ~37.6 kb when using only the 18 068 filtered SNPs to ~32.2 kb when including the genomewide SNaP markers (Table S1), and chromosome regions with low SNP density were found to be more evenly covered when SNaPs were added. Chromosomes C01 and C02, two of the *B. napus* chromosomes most substantially affected by structural rearrangements (e.g. Xiong and Pires, 2011), showed the strongest representation of SNaPs, with 1021 on C01 and 629 on C02 and an increase in haplotype blocks of 59% and 54%, respectively (Table S1).

### Genomic regions displaying SNaPs show segmental deletions in the parental lines

**Figure 2:**
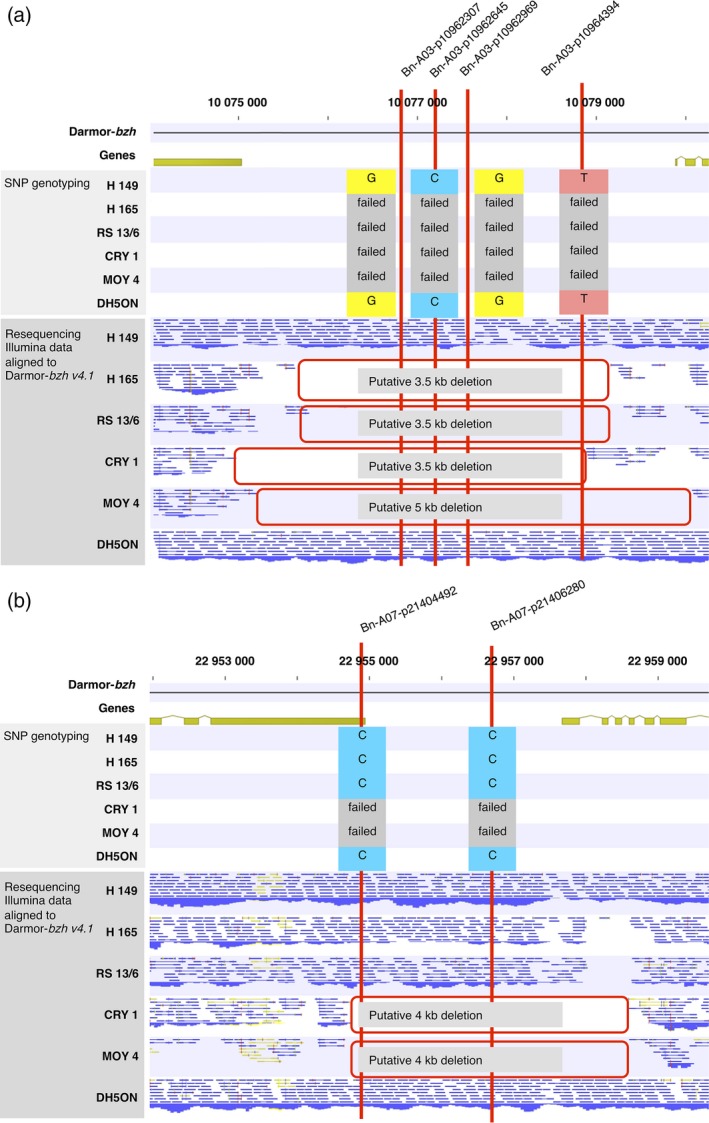
Physical anchoring of single nucleotide absence polymorphism (SNaP) markers (red lines) and Illumina resequencing data for six nested association mapping (NAM) parental lines to Darmor‐*bzh* and comparison with SNP segregation patterns in 200 NAM lines for (a) four consecutive SNaP markers on chromosome A03, Bn‐A03‐p10962307 (10 076 697 bp), Bn‐A03‐p10962645 (10 077 034 bp), Bn‐A03‐p10962969 (10 077 358 bp), Bn‐A03‐p10964394 (10 078 777 bp) and two consecutive SNaP markers on chromosome A07, Bn‐A07‐p21404492 (22 954 748 bp) and Bn‐A07‐p21406280 (22 956 683 bp).

SNaPs in a number of selected regions were validated by comparing SNP genotyping data with Illumina short‐read genomic sequence coverage from the six parental lines in the corresponding chromosome segments (genomic resequencing data from Schmutzer *et al*., 2015). The size of putatively deleted regions was manually verified and compared to genome positions of flanking sequences of the SNP assays corresponding to the respective SNaPs. Particular focus was placed on identification of small and medium scale structural variation across all chromosomes (e.g. consecutive SNaPs implying potential gene PAV). Figure 2 shows examples for deletions on chromosomes A03 and A07, respectively. Four consecutive SNaPs were detected within a 5‐kb interval on chromosome A03 from position 10 075 405 to 10 080 123 bp. Physical anchoring of these markers to the reference genome showed consecutive failed calls, and the deletion in parental lines H165, RS13/6, CRY1 and MOY4 is confirmed by the read mapping data from whole‐genome sequencing (Figure 2a). Similarly, two consecutive SNaPs on chromosome A07 exhibit failed calls in the parental lines CRY1 and MOY4, consistent with sequencing reads aligned to the reference genome (Figure 2b). From 3627 SNaPs selected using the customized filtering approach on all chromosomes, 3405 (89%) showed the corresponding SNaP between the respective parental lines. Furthermore, from 100 randomly selected SNaP markers spanning all chromosomes, we confirmed 95% putative deletions using genomewide resequencing data from the six parents.

**Figure 3:**
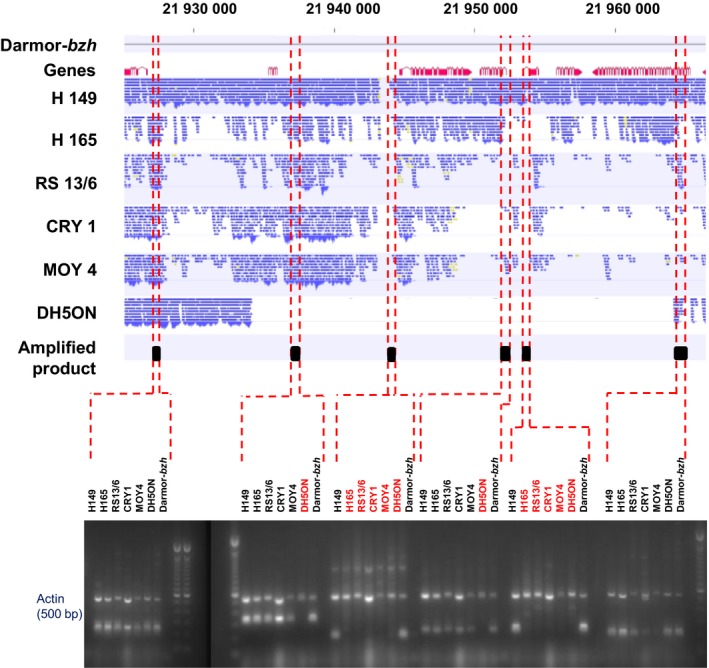
Alignment of Illumina resequencing data for six nested association mapping parents to the Darmor‐*bzh* reference and comparison with PCR amplification results for six genes contained within a 30‐kb deletion (position 21 934 109 to 21 964 245 bp on chromosome A03) in the common parent DH5ON (Actin gene and genotype Darmor‐*bzh* are used as controls, and genotypes with no amplified products are shown in red font).

Another 35‐kb deletion was detected by SNaPs in the NAM panel which was localized at position 21 935–21 965 kb on chromosome A03 in the recurrent parental line DH5ON. The SNaPs present within this region were associated with disease resistance (see below). The deletion was further validated by PCR using specific primer pairs targeting the corresponding chromosome region. As expected, DH5ON showed no amplification of specific primer pairs for four consecutive regions (containing six genes) in the estimated deleted interval, whereas the expected PCR products that indicate presence were visible in the other parental lines and positive controls (Figure 3). Based on the SNP genotyping data, this deletion was carried by 128 offspring lines of the 200 investigated NAM lines (64% frequency). A further 12‐kb deletion detected by SNaPs associated with blackleg resistance on chromosome C04 was also validated using the same approach and including Sanger sequencing of PCR products (Figure S1). The results confirmed deletion of three genes in parent MOY4 and in the respective subfamily (3.5% frequency in all 200 lines, 17.5% frequency in the subfamily).

### Deletion sizes and segregation in NAM subfamilies

**Figure 4:**
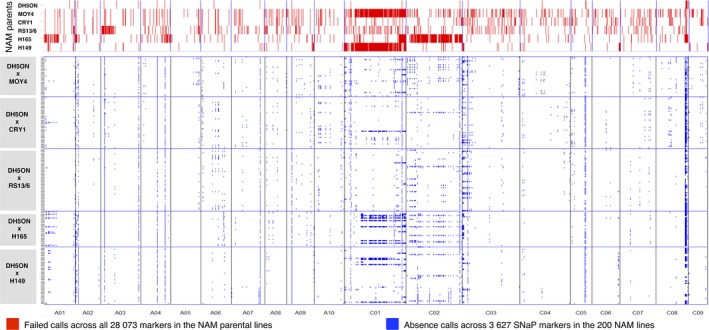
Genomewide deletion patterns visualized by single nucleotide polymorphism marker distribution with failed calls (red) in six nested association mapping (NAM) parental lines compared to single nucleotide absence polymorphism (SNaP) marker segregation patterns in the 200 *B. napus* nested association mapping (BnNAM) lines and subpopulations (blue).

Physical location of SNP probes corresponding to SNaP markers confirmed small, medium and large deletions up to chromosome‐size in the five segregating subpopulations (Figure 4). On chromosome C02, a large range deletion was detected by consecutive SNaP calls in the resynthesized *B. napus* parent H165, consistent with whole‐genome resequencing data and read coverage analysis. This deletion segregates in the DH5ON x H165 subpopulation in our study. Chromosome C01 also shows large deletions in the parents H149 and MOY4. In general, the natural *B. napus* parent DH5ON exhibits the lowest frequency of genome restructuring and SNaPs, but these segregate in all subfamilies.

### SNaP markers reveal hidden QTL for disease resistance

Genomewide SNP‐trait association analyses were first conducted using the 18 076 quality‐filtered SNP markers that were polymorphic in the NAM subpopulations. In order to reduce the rate of false‐positive marker–trait associations, a mixed linear model was applied that controls population substructure using the first two principle components and the kinship matrix. Phenotype data from blackleg disease screening in field trials in France revealed 12 significant SNP‐trait associations (higher than the arbitrary selected significance threshold of −log10_(*P*‐value)_ ≥3) with p‐values between 7.88E‐05 and 8.53E‐04 (Figure S2a). Blackleg resistance from the German field screening identified 52 significant SNP‐trait associations with p‐values higher than the arbitrary selected significance threshold (−log10_(*P*‐value)_ ≥3). After correction for false discovery rate (FDR, see experimental procedures), however, no SNPs remained in either trial with confirmed associations to blackleg disease resistance.

Repeating the GWAS including 3627 SNaPs together with the 18 076 SNPs identified a total of 38 resistance‐associated markers on the French trial data (a 3.2‐fold increase, Figure S2b, Table 1). In contrast to the SNP analysis, six associations involving SNaP markers on chromosome C04 were also significant at FDR ≤ 0.1. These identified two new QTL located in strongly conserved LD blocks from 0–40 and 200–400 kb at the proximal end of C04 (Figure S3). GWAS for blackleg resistance screening data from the German field trial revealed 115 significant associations (a 2.2‐fold increase compared to the SNP markers alone), of which 41 were also significant at FDR ≤ 0.1 (Table S3).

**Table 1:** Summary of significant marker–trait associations and QTL regions by applying GWAS for SNP markers only and for SNP plus SNaP markers

	SNP‐trait associations				QTL regions				
	SNP	SNP and SNaP	Overlapping	New	SNP	SNP and SNaP	Overlapping	New	Fold increase
Blackleg France field	12	38	12	26	7	22	6	18	3.14
Blackleg German field	52	120	55	65	14	28	13	15	2.00
SSR_AUDPC	15	16	15	1	5	8	4	4	1.60
SSR_7dai	16	17	15	2	7	11	5	6	1.57
SSR_14dai	9	12	9	3	4	11	2	9	2.75
SSR_21dai	7	20	7	13	2	7	2	5	3.50

AUDPC, area under the disease progress curve; SSR, *Sclerotinia* stem rot; dai, dai after inoculation; QTL, quantitative trait loci; SNP, single nucleotide polymorphism; SNaP, single nucleotide absence polymorphism; GWAS, genomewide association studies.

Similarly, using only SNP markers obtained with the commonly used filtering criteria, we identified 47 significant SNP‐trait associations for *Sclerotinia* stem rot resistance with p‐values over the arbitrary significance threshold. Adding the SNaP markers, a total of 65 markers (Table S2) associated with *Sclerotinia* stem rot resistance using the arbitrary selected significance threshold (a 1.4‐fold increase). SNaP markers revealing deletions inherited from the common elite parent DH5ON were associated with resistance to *Sclerotinia* stem rot resistance for the QTL present on A03 at position 21 Mb. The absence of these alleles in DH5ON was reconfirmed by SNP analysis in all parents and the BnNAM population by resequencing of parents and by PCR analyses (see details above, Figure 3). In contrast to the analysis with SNPs alone, a number of SNaP markers showed significant associations (exceeding the arbitrary selected threshold) to resistances against both pathogens.

Significant SNP‐trait associations for blackleg disease and *Sclerotinia* stem rot resistance were compared with previous studies performed in other *B. napus* mapping panels (Table S4).

### Deletions associate with both susceptibility and resistance

**Figure 5:**
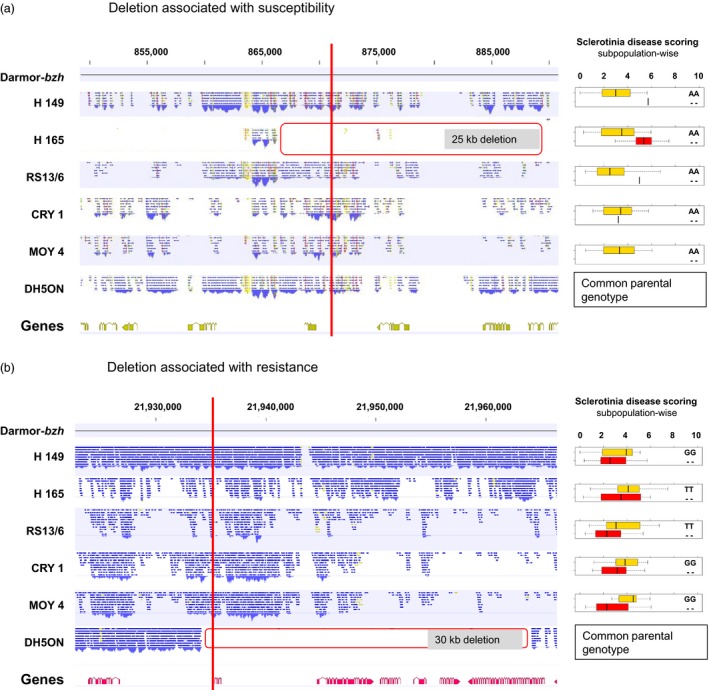
Alignment of resequencing data in six parents of the nested association mapping population showing deletions in gene range size (left, in red) compared to single nucleotide absence polymorphism marker–trait association for Sclerotinia stem rot disease resistance in five subpopulations (right). Effect of an absence allele on phenotype in the respective subfamilies showing an association with (a) susceptibility on chromosome A01 at position 873 225 bp and an association with (b) resistance on chromosome A03 at position 21 934 764 bp.

We observed opposing scenarios of PAV and its association with disease resistance. For example, an ~25 kb deletion on chromosome A01 in the parental line H165 associated with *Sclerotinia* stem rot susceptibility in the subpopulation derived from this parent (Figure 5a). In contrast, an ~30 kb deletion on chromosome A03 associated with resistance to *Sclerotinia* stem rot in all five subpopulations (Figure 5b). In general, for both diseases, absence alleles were more frequently associated with susceptibility. For blackleg, all SNaP alleles associated with susceptibility were absence alleles, while in two cases out of 30 marker–trait associations for Sclerotinia resistance a SNaP absence allele was associated with resistance.

## Discussion

SNP hybridization arrays are nowadays commonly used in genetic plant analyses (reviewed by Voss‐Fels and Snowdon, 2016). Here, we demonstrate that standard data quality filtering approaches can remove large numbers of potentially useful marker information that can mask QTL caused by PAV. We also show that these SNaP markers are detecting deletions ranging from small (1 bp) to large (chromosome‐wide) size in segregating *B. napus* populations. This has been described before using whole‐genome sequencing data for single *B. napus* genotypes (Chalhoub *et al*., 2014; Hurgobin *et al*., 2017; Samans *et al*., 2017). In the allopolyploid genome of *B. napus*, high homoeology between the diploid progenitor genomes (A and C subgenomes) led to extensive structural genome variation a widespread phenomenon in the pangenomes of many crop plants (for a review see Dolatabadian *et al*., 2017). Thus, the refined SNP filtering approach might be applicable to many agronomical important diploid and polyploidy crop genomes such as maize, sorghum, cotton wheat and others.

Using standard filtering quality procedures to select 60K Brassica Illumina SNP array markers facilitated detection of a number of regions corresponding to previously identified loci conferring disease resistance. For blackleg, studies using biparental crosses and association mapping on diverse germplasm sets have identified a small number of major loci with monogenic inheritance (Delourme *et al*., 2014; Larkan *et al*., 2014; Raman *et al*., 2012a,b) as well as multiple quantitative trait loci (QTL) involved in disease resistance (e.g. Delourme *et al*., 2006; Fomeju *et al*., 2015; Fopa Fomeju *et al*., 2014; Jestin *et al*., 2011; Kaur *et al*., 2009; Larkan *et al*., 2016). Comparison of the location of these QTL with the QTL discovered in this study is difficult as different marker types were used for genetic mapping by most authors. From the 50 QTL discovered in this study using SNP and SNaP markers, only 3 coincide with previously mapped major *Rlm* and quantitative resistance loci using SNP probe and SSR sequence anchoring to Darmor‐*bzh* (Larkan *et al*., 2014, 2016; Raman *et al*., 2012a,b, 2016). Partial resistance to *Sclerotinia* stem rot has been found in some Chinese semi‐winter *B. napus* genotypes (Zhao *et al*., 2006) and spring‐type oilseed rape lines from China and Australia (Li *et al*., 2009). QTL mapping in various Chinese DH populations has identified many loci responsible for partial resistance to *Sclerotinia* stem rot in RV298 × P1804 (Zhao *et al*., 2006), Huazhuang 5 × J7005 (Wu *et al*., 2013), Express × SWU 7 (Wei *et al*., 2014) and in natural populations by Wei *et al*. (2016) and Wu *et al*. (2016). From 37 QTL discovered in this study using SNP and SNaP markers, only seven were reported in the literature before. After adding SNaP markers, we observed a 1.6 ‐to 3.5‐fold increase of QTL regions associated with blackleg and *Sclerotinia* stem rot resistance (in total 57 new QTL were found).

Most of these previous QTL analyses included other types of markers than SNP chip markers, including anonymous amplified fragment length polymorphism or other PCR marker systems (Delourme *et al*., 2008b; Huang *et al*., 2016) that also partially address presence–absence polymorphism. Combining different marker types or marker systems has been shown to increase the power of genetic mapping because different markers access different genome features (García‐Lor *et al*., 2012; Larkan *et al*., 2014; Raman *et al*., 2012a,b). The advantages of SNP array screening, which provide robust, low‐cost, high‐resolution data for genetic mapping and GWAS, may therefore be further boosted by addition of methods to assess PAV via SNaP scoring.

Including SNaP markers in GWAS analysis for quantitative resistance to blackleg disease and *Sclerotinia* stem rot markedly increased significant marker–trait associations. The frequent localization of new QTL in regions affected by PAV confirms the hypothesis that PAV has particular relevance for disease resistance (Hurgobin *et al*., 2017). Offspring of resynthesized *B. napus* with high rates of presence–absence and other structural variations may therefore have an increased potential for use in resistance breeding of *B. napus*.

It is known that PAV and other structural variation in plants affect stress response genes, particularly genes involved in disease resistance (McHale *et al*., 2012; Shen *et al*., 2006; Tan *et al*., 2012). Examples for mutations that lead to loss of functions are numerous and have been described for many traits including disease resistance (Dolatabadian *et al*., 2017). In case of R genes, plant disease resistance is determined by complementary pairs of resistance (R) genes from the plant and avirulence (Avr) genes from the invading pathogen. In this gene‐for‐gene interaction, an avirulence protein binds to the corresponding resistance protein triggering plant defence responses. This interaction can be disrupted by a mutation in the plant R gene or in the fungal Avr gene (Bonas and Lahaye, 2002). Examples for deletions leading to a loss of function are less frequent, but have also been described. For example, presence–absence polymorphisms associated with race‐specific R genes are a common phenomenon estimated to affect about 20% of R genes in Arabidopsis and rice (Shen *et al*., 2006). Most R genes act in a dominant manner and null alleles are consequently associated with susceptibility. When grown in the absence of targeting pathogens, plants carrying specific R genes were found to be up to 10% less fit than plants lacking the R gene (MacQueen and Bergelson, 2016; Tian *et al*., 2003). Thus, deletion of R genes can be beneficial and may be a common feature of crop resistance. Association of deletions resulting in a loss of function with susceptibility is consistent with our finding that in the majority of cases the null alleles of SNaP markers were associated with *Sclerotinia* stem rot and blackleg disease susceptibility, not with resistance. However, in 6% of cases, we found a homozygous null allele of a SNaP marker associated with resistance against *Sclerotinia* stem rot. This may indicate that a plant factor allowing the pathogen to invade more efficiently is deleted or mutated. For example, Uppalapati *et al*. (2012) described a mutation of the *irg1* gene (*inhibitor of rust germ tube differentiation 1*), which is involved in wax accumulation on the leaf surface. A homozygous mutation was found to hinder the germ tubes of the Asian soybean rust and two other fungal pathogens, preventing them from developing successfully on the leaves of *Medicago truncatula*. Association of mutations or deletions with resistance in natural populations is a rare phenomenon, as loss of function is typically recessive and in heterozygous genotypes resistance can be masked by the dominant allele or by a dosage effect.

The detection of resistance‐associated deletions and a number of new genetic loci for blackleg and *Sclerotinia* stem rot resistance in this study demonstrate the usefulness of using missing data to map invisible QTL. Analyses of genes in deleted segments associated with resistance QTL is a promising new approach to deciphering the genetic basis of quantitative resistances in oilseed rape and other crop species. The described strategy for genetic mapping using SNaP markers will also be useful for dissection of major agronomical traits in molecular plant breeding of polyploid crops.

## Experimental procedures

### Plant material

A subset of a *B. napus* nested association mapping (BnNAM) population was used in this study. The BnNAM population consists of 50 genetically diverse winter *B. napus* accessions (20 exotic *B. napus*, 30 resynthesized *B. napus*) crossed with an elite doubled haploid winter‐type line (DH5ON). Each of the 50 subpopulations is composed of ≥50 doubled haploid lines per cross (where both parents are natural *B. napus*) or ≥50 single‐backcross recombinant inbred lines (BC1‐RILs) for crosses with one resynthesized *B. napus* parent (Snowdon *et al*., 2015). The present study used five BnNAM subpopulations with a total of 200 BC1‐RILs (Table 2) derived from synthetic *B. napus* founders carrying multiple quantitative resistances.

**Table 2:** Parents, genetic origin and composition of nested association mapping subpopulations used for blackleg and Sclerotinia stem rot resistance evaluation

Parental lines	Type	Accession name	Number of RILs	Mother	Variety/type	Father	Variety/type
PBY033	Synthetic	H149	48	*Brassica oleracea* conv. *capitata* var. *medullosa*	‘Cavalier rouge’	*B. rapa* ssp. *chinensis*	Pak Choi
PBY034	Synthetic	H165	28	*B. oleracea* conv. *capitata* var. *sabauda*	Wirsing	*B. rapa* ssp. *chinensis*	Pak Choi
PBY040	Synthetic	RS13/6	53	*B. rapa* ssp. *chinensis*	Pak Choi	*B. oleracea* conv. *botrytis* var. *alboglabra*	Broccoli
PBY050	Synthetic	CRY1	41	*B. rapa* spp. *trilocularis*	Yellow Sarson	*B. cretica*	–
PBY052	Synthetic	MOY4	31	*B. rapa* spp. *trilocularis*	Yellow Sarson	*B. montana*	–
PBY061	Elite	DH5ON	–	*B. napus* ssp. *napus*	Oase	*B. napus* ssp. *napus*	Nugget

### Phenotypic analysis of traits

Blackleg resistance testing of the 200 BnNAM accessions was conducted in a field screening by Syngenta (Toulouse, France) and KWS SAAT SE (Einbeck, Germany) in 2015/2016. The area of necrosis at the plants base stem was evaluated for 30 plants per genotype using a 1–6 scale at crop maturity in late June. This procedure is also known as the G2 index, where a score of 1 corresponds to complete absence of affected tissue, while a score of 6 corresponds to 100% area affected, a broken stem or a dead plant (Delourme *et al*., 2008a,b; Huang *et al*., 2016). *Sclerotinia* stem rot resistance tests were conducted in a field screening at KWS SAAT SE, Einbeck, Germany, in 2015/2016. Resistance was assessed using a toothpick stem inoculation method similar to the method described by Zhao and Meng (2003). Plants in plots were inoculated after flowering with toothpicks that were previously soaked with potato dextrose broth and overgrown with *S. sclerotiorum* mycelia. The toothpicks were inserted into the centre of the main stem and the lengths of necrotic surface were measured at 7, 14 and 21 days after inoculation (dai). Approximately 25 plants were scored for each of the 200 tested BnNAM lines. Using the lesion lengths recorded at the three dates, an area under the disease progress curve (AUDPC) was calculated according to Obermeier *et al*. (2013). Mean values for each of the three time points (7, 14, 21 dai) and the AUDPC values were used for GWAS.

### SNP genotyping and quality control

The entire BnNAM panel was genotyped with the 60K Illumina Infinium Brassica SNP array containing 52 158 SNP probes. Using the Darmor‐*bzh* reference v4.1 (Chalhoub *et al*., 2014), we anchored 28 073 SNP marker using BLASTN as described by Qian *et al*. (2014). Initially, all markers that exhibited a minor allele frequency (MAF) <0.05 and a failed call frequency >90% were removed from the SNP data set. Subsequently, SNP markers that were previously anchored to the Darmor‐*bzh* reference v4.1 were included in a customized pipeline, regardless of whether they were polymorphic or monomorphic for expected SNP alleles, to evaluate whether they show segregation patterns consistent with a presence–absence polymorphism (SNaPs), indicating a possible deletion. If a SNP exhibited a presence–absence segregation pattern ratio in at least one of the subfamilies, the presence SNP allele was recoded as AA and the failed SNP call as BB to enable inclusion in the marker matrix for the GWAS.

### Genomewide association studies, linkage disequilibrium analysis and haplotype construction

Association analyses were conducted using the R package GenABEL (Aulchenko *et al*., 2007). A mixed linear model approach that increases detection power (Stich *et al*., 2008) was adjusted for population stratification by including the kinship matrix and the first two principal components as covariates (Price *et al*., 2006). For determining significant SNP‐trait association, we applied a FDR of ≤0.1 (Benjamini and Hochberg, 1995). Additionally, a significance cut‐off value was set at −log_10_(1/*n*), where *n* represents the number of SNP markers. To reduce the type II error rate, we also captured the SNP‐trait associations for disease resistance using an arbitrary threshold of −log_10(*P*‐value)_ ≥3 as previously done by Hatzig *et al*. (2015) and Raman *et al*. (2016). Whole‐genome linkage disequilibrium was calculated using the squared allele frequency correlations (*r* ^2^) between pairs of SNPs. Only markers with MAF ≥ 0.05 were included in the analysis. Haplotype patterns were assessed for SNPs and SNaPs that showed significant marker trait association. Haplotype blocks were defined using the confidence interval method described by Gabriel *et al*. (2002) in Haploview version 4.2 (Barrett *et al*., 2005) and the R package GenABEL (Aulchenko *et al*., 2007).

### PCR validation of PAV

Specific primers were designed for all genes present in QTL intervals that showed significant associations between SNaP markers and traits on chromosomes A03 and C04. Additionally, primers specific for an *Actin* gene copy located on C04 (BnaC04g27010D) were designed and included in a multiplex PCR to ensure that no technical errors occurred during the tests. Additionally, we included for PCR the *B. napus* reference genotype Darmor‐*bzh* as a positive control for PCR amplification. PCR primer information, reaction setup and conditions are listed in Table S5.

### Comparative sequence analysis of QTL regions from six NAM parents

The publically available *B. napus* Darmor‐*bzh* reference genome assembly v. 4.1 (Chalhoub *et al*., 2014) and the resequencing data sets of the six BnNAM parents with a 12 to 15× coverage of Illumina 100‐bp paired‐end‐sequencing (Schmutzer *et al*., 2015) were used for comparative analysis of read coverage and PAV in selected QTL regions. Illumina sequence reads of the six parental lines were aligned to the reference genome with CLC Genomics Workbench v.9.0 software (Qiagen Bioinformatics, Aarhus, Denmark). Putative PAVs were visually inspected using CLC Genomics Workbench. To remove putative false‐positive aligned short Illumina reads, a minimum threshold of five reads aligned to a physical position in the reference was set, similar to the approach described by Schmutzer *et al*. (2015).

## Conflict of interest

The authors declare no conflicts of interest.

## Author contributions

IG, RS and CO designed the concept and wrote the manuscript. IG and CO and analysed the data. IG, CO, XL and HSC performed the PCR analyses. VK, RD, AvT, SF, CJ, ED, SV and FB contributed to phenotype data acquisition. All authors read and approved the manuscript.

## Supporting information


**Figure S1.** Sequence analyses for a QTL detected for blackleg disease resistance in DH line MOY4, covering a 12 kb region on chromosome C04 (C04_QTL1). (a) Anchoring of consensus Sanger reads to the reference genome Darmor‐*bzh*; (b) anchoring of Sanger reads to individual NAM parents of five targeted genes; (c) PCR amplification of genes.

**Figure S2.** Manhattan plots resulting from genome‐wide association analysis (GWAS) for blackleg resistance in the NAM panel using (a) SNP markers, (b) SNP and SNaP markers. The *x*‐axis represents the marker positions along each chromosome anchored to the Darmor‐*bzh* reference; the *y*‐axis shows the −log10_(*P*‐value)_ for the trait–marker association. The solid horizontal line indicates the arbitrary selected threshold at −log10_(*P*‐value)_ ≥3 and the dashed line indicates the significance threshold −log10_(*P*‐value)_ ≥4.33 or FDR <0.10.

**Figure S3.** Detection of a QTL for blackleg disease resistance on chromosome C04 using GWAS with (a1) only SNP markers, and (a2) SNP plus SNaP markers. Haplotype patterns reveal two blocks at the beginning of the chromosome, one (BnPAV_C04_1) spanning 40k and harbouring 3 SNaP markers (b1), and 13 genes (c1), and a second (BnPAV_C04_2) spanning 200k and harbouring 2 SNaP markers (b2), and 19 genes (c2), respectively.

**Table S1.** Mean genomic distances and haploblock numbers between SNP markers and between SNP and SNaP markers, respectively.
**Table S2.** Summary of SNP‐trait associations with a −log10_(*P*‐value)_ ≥3.
**Table S3.** Summary of SNP‐ and SNaP‐trait associations with a −log10_(*P*‐value)_ ≥3.
**Table S4.** Common QTL regions with literature.
**Table S5.** Information on PCR primer sequences, reaction setups and cycling conditions.
