# Supplementary material for: Finding invisible quantitative trait loci with missing data
Source: Plant Biotechnol J. 2018 May 28;16(12):2102–12. doi: 10.1111/pbi.12942 (PMC6230954; doi:10.1111/pbi.12942)
Supplement: Supplementary file 1 — Figure S1. Sequence analyses for a QTL detected for blackleg disease resistance in DH line MOY4, covering a 12 kb region on chromosome C04 (C04_QTL1). (a) Anchoring of consensus Sanger reads to the reference genome Darmor‐bzh; (b) anchoring of Sanger reads to individual NAM parents of five targeted genes; (c) PCR amplification of genes. [file PBI-16-2102-s003.pdf]

Supplementary figures

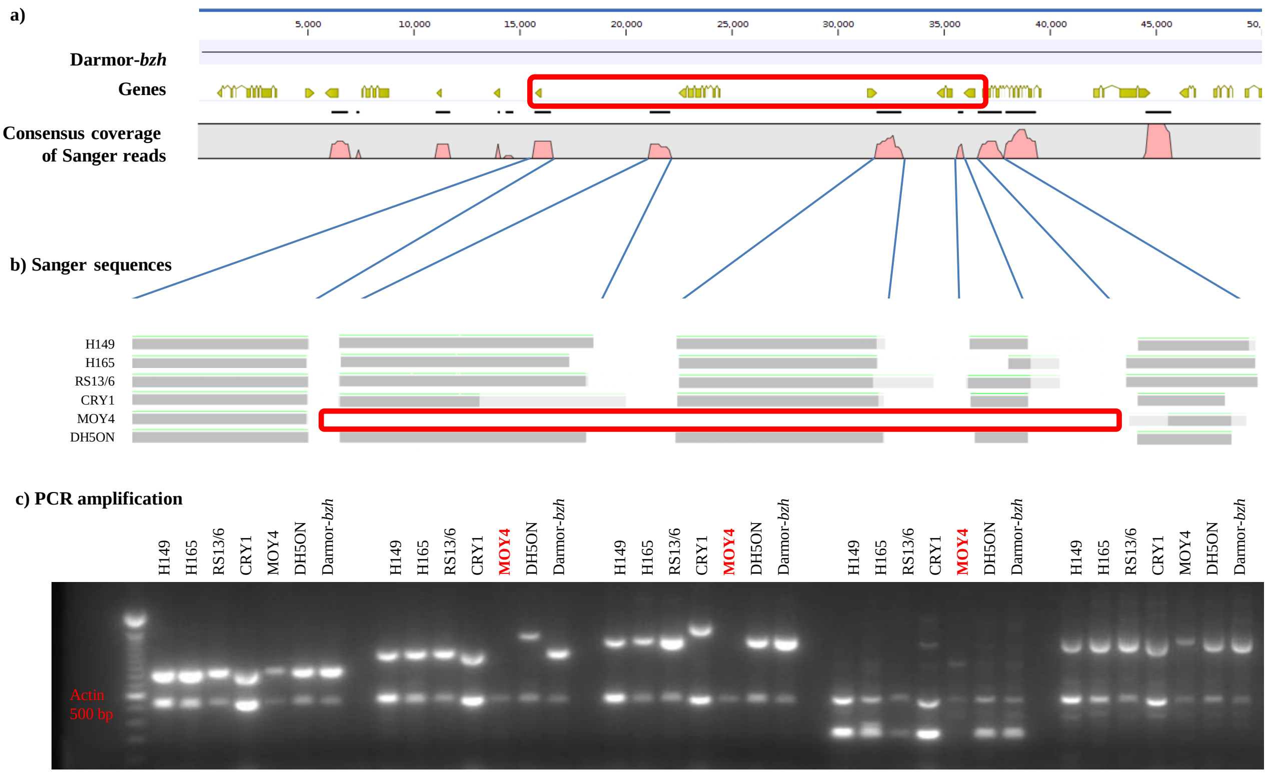

Figure S1 Sequence analyses for a QTL interval detected for blackleg disease resistance in line MOY4, covering a 12 kb region on chromosome C04 (C04_QTL1). a) anchoring of consensus Sanger reads to the reference genome Darmor-*bzh*; b) anchoring of Sanger sequencing to individual NAM parents of five targeted genes; c) PCR amplification of genes.
