# Supplementary material for: Finding invisible quantitative trait loci with missing data
Source: Plant Biotechnol J. 2018 May 28;16(12):2102–12. doi: 10.1111/pbi.12942 (PMC6230954; doi:10.1111/pbi.12942)
Supplement: Supplementary file 2 — Figure S2. Manhattan plots resulting from genome‐wide association analysis (GWAS) for blackleg resistance in the NAM panel using (a) SNP markers, (b) SNP and SNaP markers. The x‐axis represents the marker positions along each chromosome anchored to the Darmor‐bzh reference; the y‐axis shows the −log10(P‐value) for the trait–marker association. The solid horizontal line indicates the arbitrary selected threshold at −log10(P‐value) ≥3 and the dashed line indicates the significance threshold −log10(P‐value) ≥4.33 or FDR <0.10. [file PBI-16-2102-s002.pdf]

Supplementary figures

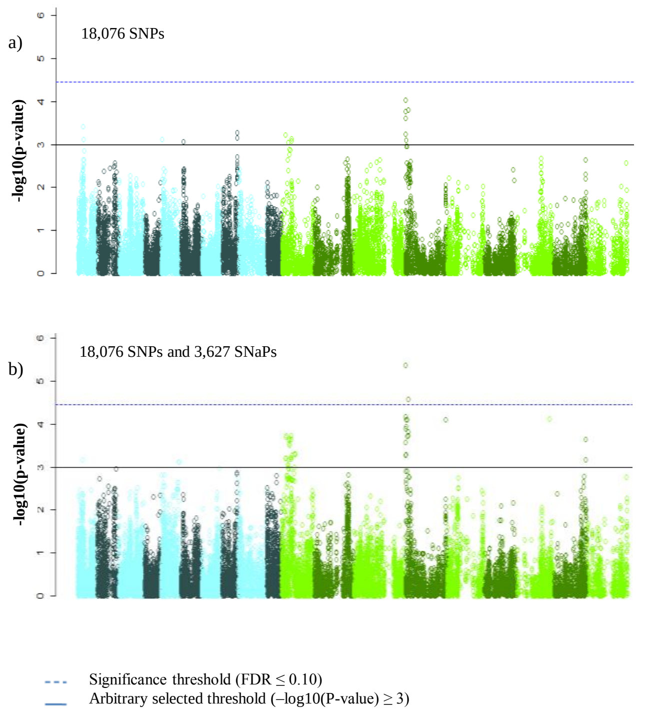

Figure S2 Manhattan plots resulting from genome-wide association analysis (GWAS) for Blackleg resistance in the NAM panel using (a) SNP markers, (b) SNP and SNaP markers. The x-axis represents the marker positions along each chromosome anchored to the Darmor-*bzh* reference; the y-axis shows the -log10(P-value) for the trait-marker association. The solid horizontal line indicates the arbitrary selected threshold at -log10(P-value) ≥ 3 and the dashed line indicates the significance threshold –log10(P-value) ≥ 4.33 or FDR < 0.10.
