# Supplementary material for: Finding invisible quantitative trait loci with missing data
Source: Plant Biotechnol J. 2018 May 28;16(12):2102–12. doi: 10.1111/pbi.12942 (PMC6230954; doi:10.1111/pbi.12942)
Supplement: Supplementary file 3 — Figure S3. Detection of a QTL for blackleg disease resistance on chromosome C04 using GWAS with (a1) only SNP markers, and (a2) SNP plus SNaP markers. Haplotype patterns reveal two blocks at the beginning of the chromosome, one (BnPAV_C04_1) spanning 40k and harbouring 3 SNaP markers (b1), and 13 genes (c1), and a second (BnPAV_C04_2) spanning 200k and harbouring 2 SNaP markers (b2), and 19 genes (c2), respectively. [file PBI-16-2102-s004.pdf]

Supplementary figures

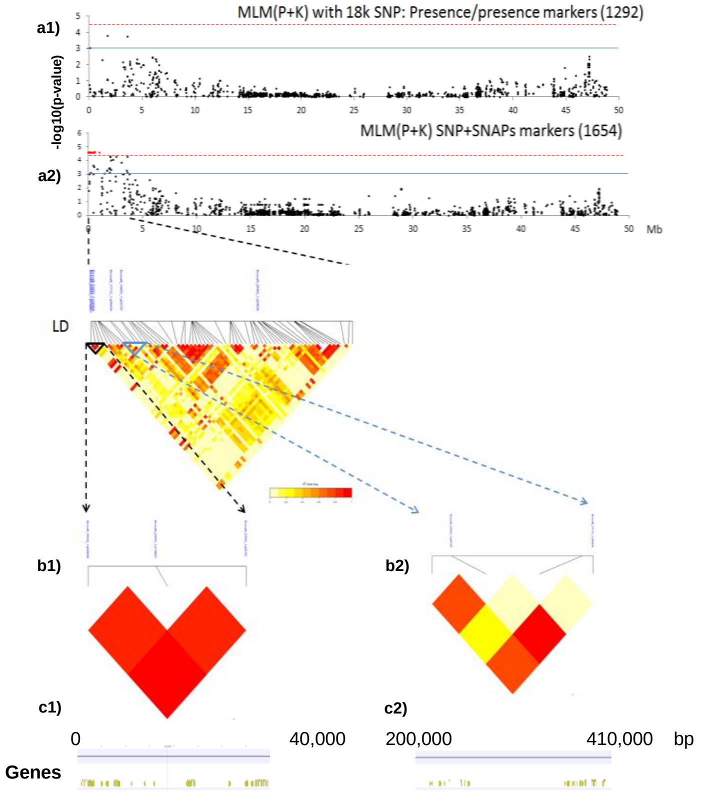

Figure S3 Detection of a QTL for blackleg disease resistance on chromosome C04 using GWAS with (a1) only SNP markers, and (a2) SNP plus SNaP markers. Haplotype patterns reveal two blocks at the beginning of the chromosome, one (BnPAV_C04_1) spanning 40 kb and harbouring 3 SNaP markers (b1), and 13 genes (c1), and a second (BnPAV_C04_2) spanning 200 kb and harbouring 2 SNaP markers (b2), and 19 genes (c2), respectively.
